# Association between fresh frozen plasma transfusion and mortality stratified by Glasgow Coma Scale scores in isolated traumatic brain injury: a nationwide cohort study in Japan

**DOI:** 10.1007/s00068-026-03249-7

**Published:** 2026-07-01

**Authors:** Tatsunori Nagamura, Makoto Aoki, Kohei Yamada, Takero Terayama, Soichiro Seno, Satoshi Tomura, Tetsuro Kiyozumi

**Affiliations:** 1 Department of Traumatology and Critical Care Medicine, National Defense Medical College, Saitama, Japan; 2 Division of Traumatology, Research Institute, National Defense Medical College, Saitama, Japan; 3 Department of Emergency and Critical Care Medicine, Japan Self-Defense force Central Hospital, Tokyo, Japan

**Keywords:** Traumatic brain injury, Fresh frozen plasma, Glasgow Coma Scale, Coagulopathy

## Abstract

**Purpose:**

Fresh frozen plasma (FFP) is widely used to manage trauma-induced coagulopathy. However, its effectiveness in isolated traumatic brain injury (TBI) management remains controversial, particularly when stratified by severity based on the Glasgow Coma Scale (GCS) scores. This study aimed to examine the association between FFP administration within 24 h and mortality in patients with isolated TBI, overall and stratified by GCS on arrival.

**Methods:**

We conducted a multicenter retrospective cohort study using the Japan Trauma Data Bank (2019–2023). Adults with isolated TBI (intracranial AIS ≥ 3 and extracranial AIS < 3) were included. Patients were classified into FFP and non-FFP groups based on transfusion within 24 h of admission. Subgroup analyses were performed by TBI severity (severe: GCS ≤ 8; mild-to-moderate: GCS > 8), craniotomy status, and FFP dose. The primary outcome was 28-day mortality. Propensity score matching was applied to adjust for confounding.

**Results:**

Among 12,480 patients (median age 72 years [IQR: 55, 82]; 66.6% male), 513 received FFP. After matching, FFP was not significantly associated with 28-day mortality (OR 0.80; 95% CI 0.59–1.08). In subgroup analyses, FFP was associated with higher mortality in mild-to-moderate TBI (OR 2.38; 95% CI 1.07–5.31) and lower mortality in severe TBI (OR 0.61; 95% CI 0.42–0.88). No significant differences were observed by craniotomy status or FFP dose.

**Conclusions:**

FFP administration was not associated with lower 28-day mortality in isolated TBI. Although subgroup analyses suggested the association between FFP administration and outcomes may vary according to TBI severity, these findings should be interpreted cautiously given the potential for residual confounding.

**Supplementary Information:**

The online version contains supplementary material available at 10.1007/s00068-026-03249-7.

## Introduction

Traumatic brain injury (TBI) is a leading cause of death and disability worldwide. An estimated 20.84 million new TBI cases were reported globally in 2021, underscoring the substantial burden this condition places on healthcare systems [1]. Despite significant advances in trauma care and neurosurgical management, the mortality rate among patients with severe TBI remains high at 27.8% [2].

Coagulopathy in TBI has been reported to occur in 13–54% of patients [3] and in more than 60% of patients with severe TBI [4]. Fresh frozen plasma (FFP) is widely used to manage coagulopathy in patients with trauma. In patients with isolated TBI, FFP is often used for correction of coagulopathy or INR reversal, and in some cases may be administered preemptively prior to neurosurgical procedures, particularly in Japanese neurotrauma care. However, its effectiveness in isolated TBI remains controversial. A single-center retrospective study reported that achieving a serum fibrinogen level ≥ 1.5 g/L 3 h after FFP administration was associated with higher 3-month GOS [5]. Another study demonstrated that early plasma transfusion was associated with improved in-hospital survival in a subgroup of patients with multicompartmental intracranial hemorrhage [6]. In contrast, a large analysis from the Trauma Quality Improvement Program (TQIP) reported a potential association between higher FFP volume and increased mortality [7]. Despite its widespread use, it remains unclear whether FFP administration is associated with improved outcomes in patients with isolated TBI. In addition, most previous studies have focused on severe TBI, and data on mild-to-moderate TBI are limited. Therefore, we aimed to investigate the association between FFP administration within 24 h and mortality in patients with isolated TBI using data from a nationwide trauma registry.

## Methods

### Study design, setting, and data source

This retrospective cohort study used data from the Japan Trauma Data Bank (JTDB) for the period 2019 to 2023. The JTDB is a nationwide hospital-based trauma registry in Japan, established in 2003 by the Japanese Association for the Surgery of Trauma (Trauma Registry Committee) and the Japanese Association for Acute Medicine (Committee for Clinical Care Evaluations) [8, 9]. In total, 291 emergency medical institutions across Japan participated in the registry in 2024, including 95% of tertiary emergency medical centers in the country [10]. This study adhered to the Strengthening the Reporting of Observational Studies in Epidemiology (STROBE) guidelines [11]. The study was approved by the institutional review board of the National Defense Medical College (approval number: 4971), which waived the requirement for informed consent owing to the retrospective observational study design and the use of existing medical information.

### Study participants

The study included patients (aged ≥ 16 years) with isolated TBI who were identified by an intracranial Abbreviated Injury Scale (AIS) score of ≥ 3 and an extracranial AIS score of < 3, according to previous studies [12–14]. The exclusion criteria were as follows: (1) transfer from another hospital, (2) past history of antithrombotic therapy, (3) prehospital cardiopulmonary arrest, (4) cardiopulmonary arrest upon hospital arrival, (5) AIS score of 6 for any region, (6) receipt of red blood cell (RBC) transfusion within 24 h of arrival at the hospital, and (7) past history of moderate to severe liver disease. Patients who received RBC transfusions were excluded because FFP is often administered as a component of a balanced transfusion rather than specifically for TBI-related coagulopathy, and the inclusion of RBCs could introduce bias and obscure the independent effects of FFP. Moreover, it is closely linked to traumatic hemorrhage and injury severity, which could lead to the inclusion of confounding factors in the mortality assessment [15].

### Study variables

The following variables were collected: sex; Charlson comorbidity index (CCI); injury type; intracranial hematoma; vital signs on hospital arrival; Glasgow Coma Scale (GCS) score; systolic blood pressure (SBP); heart rate (HR); TBI type; head AIS score; injury severity score (ISS); tranexamic acid (TXA) use; blood transfusion including red blood cell (RBC); FFP within 24 h of arrival; craniotomy; and mortality. Craniotomy in this study referred to open cranial surgical interventions for intracranial hemorrhage, including craniotomy and decompressive craniectomy, but excluding burr hole procedures and endovascular interventions, as recorded in the JTDB. Injury type referred to the mechanism of trauma, such as traffic accidents and falls, based on a previous study [16]. TBI was further categorized as hemorrhagic TBI (epidural hematoma [EDH], subdural hematoma [SDH], subarachnoid hemorrhage [SAH], and intraparenchymal hemorrhage [IPH]) and non-hemorrhagic TBI (including diffuse axonal injury, diffuse brain swelling, and selected cases of pneumocephalus meeting AIS ≥ 3 criteria); therefore, simple concussion without intracranial injury was not included. Hemorrhagic TBI was classified according to the AIS codes outlined in Supplementary Table 1 [17].

### Exposure and primary outcome measure

Patients who underwent FFP transfusion (FFP group) were compared with those who did not (non-FFP group). The primary outcome was the 28-day mortality rate. The secondary outcomes selected a priori were the 24-h mortality rate, in-hospital mortality rate, and overall complications. Complications included pulmonary edema, pulmonary embolism, acute respiratory distress syndrome (ARDS), pneumonia, acute kidney injury, and sepsis.

### Statistical analysis

Continuous and categorical variables are reported as medians with interquartile ranges (IQR) and count with percentages, respectively. The chi-square test was used for categorical data and the Mann–Whitney U test for continuous data to compare the clinical characteristics of patients between the FFP and non-FFP groups. Missing data were imputed using the *missForest* package in R, which provides a single imputation based on nonparametric random forest algorithms [18]. This method does not generate multiple datasets; however, it has been shown to achieve superior performance in managing complex interactions and nonlinear relationships in clinical datasets [19, 20]. The variables imputed included sex (1.1% missing), CCI (0.03% missing), transfer from other hospitals (2.3% missing), history of anticoagulant use (0.2% missing), SBP measured during prehospital care (18.6% missing), SBP on arrival (1.1% missing), HR on arrival (1.6% missing), GCS score on arrival (2.5% missing), ISS (0.3% missing), and alcohol use (31.8% missing). Age, sex, CCI, injury type, intracranial hematoma, vital signs on arrival, head AIS score, ISS, TXA transfusion, craniotomy, and alcohol use were used as factors to predict the propensity scores for FFP treatment by using multivariate logistic regression analysis in accordance with previous studies [7, 15]. We included craniotomy as a confounding factor because FFP administration in Japanese clinical practice may occur after the clinical decision to perform a craniotomy, necessitating adjustment for injury severity. In addition, the proportion of patients undergoing craniotomy differed markedly between the FFP and non-FFP groups, further justifying its inclusion in the model to account for this imbalance and minimize severity-related confounding.

Using a caliper of 0.2, propensity score matching (PSM) extracted 1:1 matched pairs of patients based on propensity scores. Before and after PSM, the distribution of propensity scores between the FFP and non-FFP groups was examined. The match balance between the two groups was assessed using the standardized mean difference (SMD), and an acceptable balance was defined as an SMD of < 0.1 [21].

First, standard logistic regression was applied to the matched dataset to estimate the odds ratios (ORs) for clinical outcomes, including 28-day and 24-h mortality rates and complications. Second, subgroup analyses were performed to explore potential candidates who would benefit from FFP transfusions. To ensure statistical stability and minimize bias in small subsets, we adopted an ‘across-subsets’ strategy as recommended by recent methodological literature [22]. Specifically, propensity scores were estimated using an overall model, and subgroup analyses were subsequently conducted on the matched dataset. Subgroups were stratified by (1) TBI severity based on GCS (GCS score ≤ 8: severe and GCS score ≥ 9: mild to moderate) [23], (2) hemorrhagic versus non-hemorrhagic TBI, (3) presence or absence of craniotomy, and (4) FFP transfusion volume (divided into high and low groups using the mean volume as the cutoff). Patients in the mild-to-moderate subgroup were divided into mild (GCS 13–15) and moderate (GCS 9–12), and logistic regression with an interaction term was used to assess differences in the association between FFP and outcomes between these groups. To account for potential covariate imbalances and evaluate treatment effect heterogeneity, we additionally performed multivariable logistic regression including interaction terms between FFP and each subgroup variable.

Kaplan–Meier survival curves were generated to compare 28-day survival between the FFP and non-FFP groups in the propensity score-matched cohort. Differences in survival distribution were assessed using the log-rank test. This analysis was performed for the overall matched cohort, as well as separately for subgroups stratified by TBI severity: severe and mild to moderate.

A sensitivity analysis was conducted to assess the robustness of the findings. First, to evaluate the potential impact of patients who received RBC transfusion, we repeated the propensity score–matched analysis in a broader cohort that included patients who had received RBC transfusion within 24 h. In this analysis, RBC transfusion was incorporated as an additional covariate in the propensity score model alongside the original covariates. Second, to address potential immortal time bias, we performed another propensity score–matched analysis excluding patients who died within 24 h of hospital arrival, using the same covariate set as in the primary model.

Statistical significance was defined as a two-sided p-value of < 0.05 or assessed using a 95% confidence interval (CI). We defined statistical significance in the interactions within subgroup analysis as p-values for interactions of less than 0.1 [24]. All statistical analyses were performed using EZR (version 2.4.2; Japan).

## Results

**Fig. 1:**
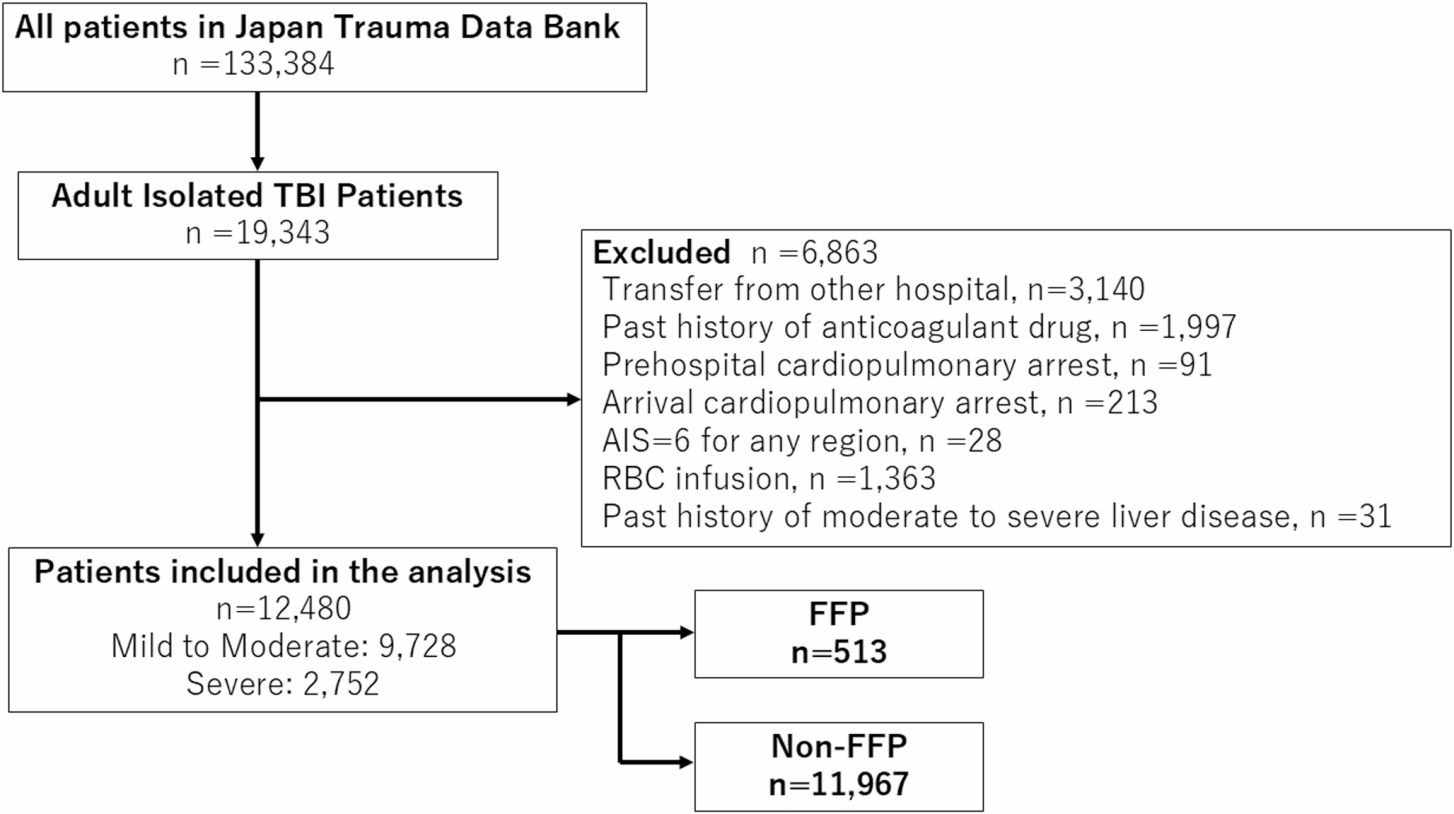
Flow diagram of the study participant selection process. TBI, traumatic brain injury; AIS, Abbreviated Injury Scale; FFP, fresh frozen plasma

A total of 133,384 trauma patients were registered in the JTDB during the study period. After applying the inclusion and exclusion criteria, 12,480 patients with isolated TBI were included in the final analysis, of whom 513 (4.1%) received FFP within 24 h of hospital arrival (Fig. 1). The cohort was predominantly elderly (median age 72 years [IQR: 55, 82]) and largely composed of patients with mild-to-moderate TBI (77.9%). The overall 28-day mortality rate was 11.4%. Among patients who received FFP, the median transfusion volume was 4 units [IQR: 4, 8] (see Supplementary Table 2 for full baseline characteristics).

Before matching, patients who received FFP had a more severe clinical profile, indicating greater neurological impairment and overall injury burden. After propensity score matching, baseline characteristics were well balanced between groups, supporting the validity of subsequent comparisons (Table 1 and Supplementary Fig. 1).

**Table 1:** Characteristics of patients before and after propensity score matching

	Total patients			PSM patients		
	FFP group *n* = 513	Non-FFP group *n* = 11,967	ASMD	FFP group *n* = 506	Non-FFP group *n* = 506	ASMD
Age, y, median (IQR)	63 [43, 77]	72 [56, 82]	0.38	63 [43, 77]	67 [44, 79]	0.09
Sex, male, n (%)	388 (75.6)	7,921 (66.2)	0.21	381 (75.3)	370 (73.1)	0.05
Charlson score, median (IQR)	0 [0, 0]	0 [0, 1]	0.18	0 [0, 0]	0 [0, 0]	< 0.01
Alcohol use, n (%)	177 (34.5)	3,431 (28.1)	0.13	175 (34.6)	175 (34.6)	< 0.01
Injury type, n (%)						
Motor vehicle crash	19 (3.7)	449 (3.8)	< 0.01	19 (3.8)	17 (3.4)	0.02
Motorcycle	64 (12.5)	704 (5.9)	0.23	63 (12.5)	56 (11.1)	0.04
Pedestrian	63 (12.3)	899 (7.5)	0.16	60 (11.9)	64 (12.6)	0.02
Bicycle	85 (16.6)	1,184 (9.9)	0.20	84 (16.6)	81 (16.0)	0.02
Falling from height	42 (8.2)	605 (5.1)	0.13	41 (8.1)	46 (9.1)	0.04
Falling at grounding level	94 (18.3)	4,748 (39.7)	0.48	94 (18.6)	92 (18.2)	0.01
Head Injury Type						
Epidural hematoma	102 (19.9)	1,229 (10.3)	0.27	99 (19.6)	94 (18.6)	0.03
Subdural hematoma	340 (66.3)	7,839 (65.5)	0.02	334 (66.0)	334 (66.0)	< 0.01
Subarachnoid hemorrhage	105 (20.5)	685 (5.7)	0.45	102 (20.2)	93 (18.4)	0.05
Intraparenchymal hematoma	23 (4.5)	332 (2.8)	0.09	23 (4.5)	25 (4.9)	0.02
SBP, mmHg, median (IQR)	151 [133, 171]	150 [131, 173]	0.03	151 [133, 170]	150 [133, 172]	< 0.01
HR, bpm, median (IQR)	86 [72, 100]	84 [73, 96]	0.01	86 [72, 101]	88 [76, 103]	0.08
GCS, median (IQR)	9 [6, 13]	14 [10, 15]	0.76	9 [6, 13]	9 [4, 14]	0.08
Head AIS, median (IQR)	4 [4, 5]	3 [3, 4]	0.74	4 [4, 5]	4 [4, 5]	0.08
ISS, median (IQR)	21 [16, 25]	14 [9, 20]	0.79	21 [16, 25]	25 [16, 24]	0.08
TXA, n (%)	269 (52.4)	2916 (24.4)	0.60	262 (51.8)	281 (55.5)	0.08
Craniotomy, n (%)	186 (36.3)	923 (7.7)	0.73	179 (35.4)	172 (34.0)	0.03

FFP, fresh frozen plasma; ASMD, absolute standardized mean difference; SBP, systolic blood pressure; HR, heart rate; GCS, Glasgow coma scale; AIS, abbreviated injury scale; ISS, injury severity scale, TXA, tranexamic acid

In the matched cohort, FFP administration was not associated with 28-day mortality (19.0% vs. 22.7%; OR 0.80, 95% CI 0.59–1.08). Although FFP use was associated with lower 24-hour mortality, this apparent short-term benefit did not persist, with no improvement in longer-term survival. In contrast, overall complication rates were higher in the FFP group (Table 2).

**Table 2:** Outcomes after propensity score matching

Outcomes	FFP *n* = 506	Non-FFP *n* = 506	OR (95%CI)	*P*-value
Primary outcome				
Mortality at 28 days, n (%)	96 (19.0)	115 (22.7)	0.80 (0.59–1.08)	0.14
Secondary outcomes				
Mortality at 24 h, n (%)	25 (4.9)	44 (8.7)	0.55 (0.33–0.91)	0.02
In-hospital mortality, n (%)	108 (21.3)	121 (23.9)	0.86 (0.64–1.17)	0.33
Overall complications, n (%)	227 (44.9)	177 (35.0)	1.51 (1.17–1.95)	0.01
Pulmonary edema, n (%)	1 (0.2)	0 (0)	Not estimable	1.00
Pulmonary embolism, n (%)	2 (0.4)	1 (0.2)	2.00 (0.18–22.2)	0.57
ARDS, n (%)	2 (0.4)	1 (0.2)	2.00 (0.18–22.2)	0.57
Pneumonia, n (%)	1 (0.2)	0 (0)	Not estimable	1.00
Acute Kidney Injury, n (%)	0 (0)	2 (0.4)	Not estimable	1.00
Sepsis, n (%)	2 (0.4)	0 (0)	Not estimable	1.00

FFP, fresh frozen plasma; OR, odds ratio; CI, confidence interval; ARDS, Acute Respiratory Distress Syndrome

**Fig. 2:**
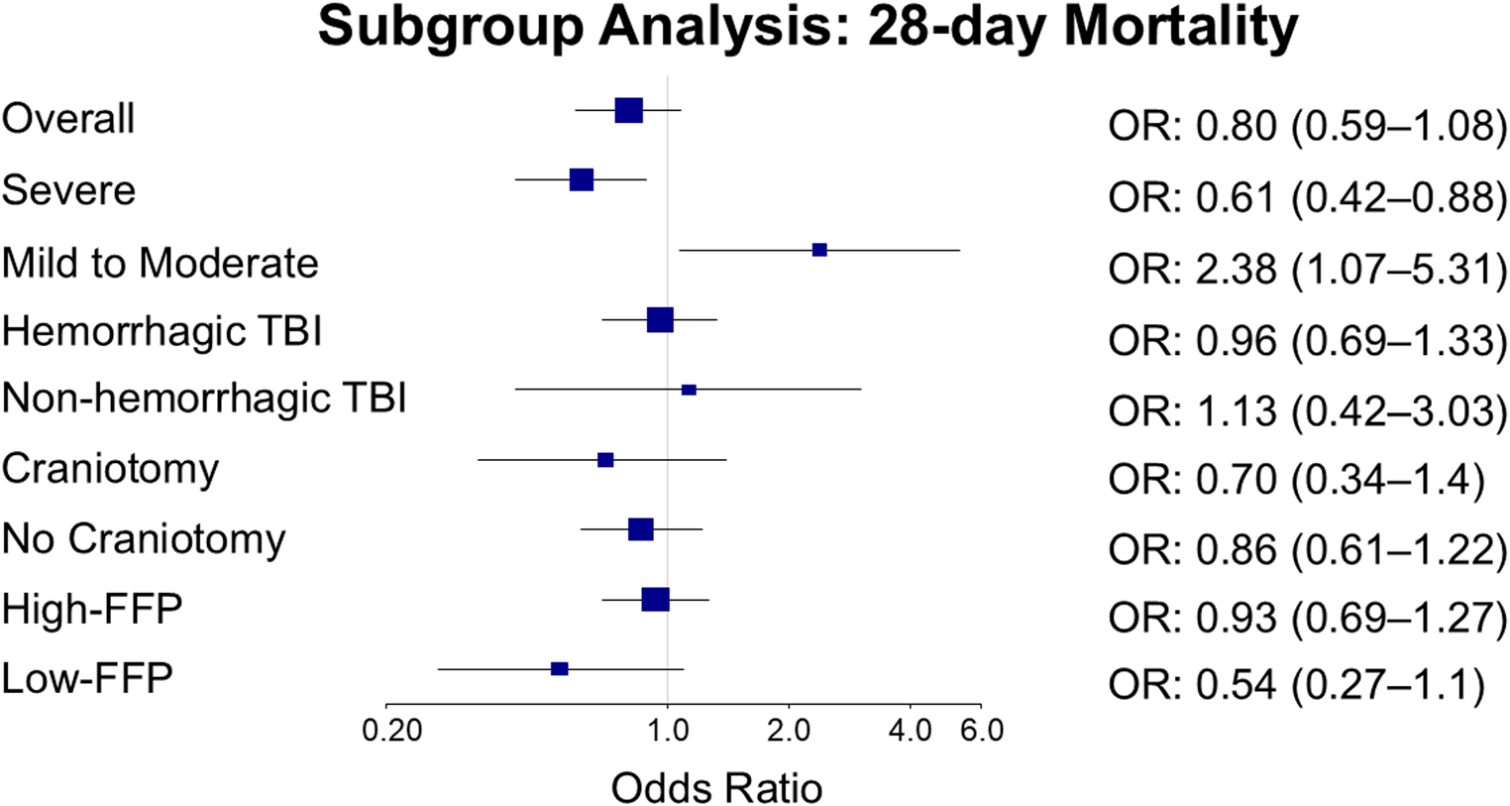
Effect of FFP administration on the 28-day mortality rate among overall and in subgroups. FFP, fresh frozen plasma

The association between FFP administration and mortality varied by TBI severity (Fig. 2). In patients with mild-to-moderate TBI, FFP administration was associated with an increased risk of 28-day mortality (OR 2.38, 95% CI 1.07–5.31). In contrast, in patients with severe TBI, FFP administration was associated with significantly lower 28-day mortality (OR 0.61, 95% CI 0.42–0.88), with a statistically significant interaction (p for interaction < 0.01). When mild and moderate TBI were analyzed separately, neither subgroup showed a statistically significant increase in mortality (mild: OR 2.72, 95% CI 0.69–10.7; moderate: OR 2.84, 95% CI 0.90–8.97). A similar pattern was observed in the hemorrhagic TBI subgroup (SDH, EDH, SAH, and IPH). While no overall association was observed, FFP administration was associated with increased mortality in the mild-to-moderate subgroup (OR 3.60, 95% CI 1.31–9.87, *p* = 0.01), but not in the severe subgroup (OR 0.79, 95% CI 0.54–1.18, *p* = 0.25), with a significant interaction between subgroups (p for interaction < 0.01). No meaningful differences were observed according to craniotomy status or FFP dose.

**Fig. 3:**
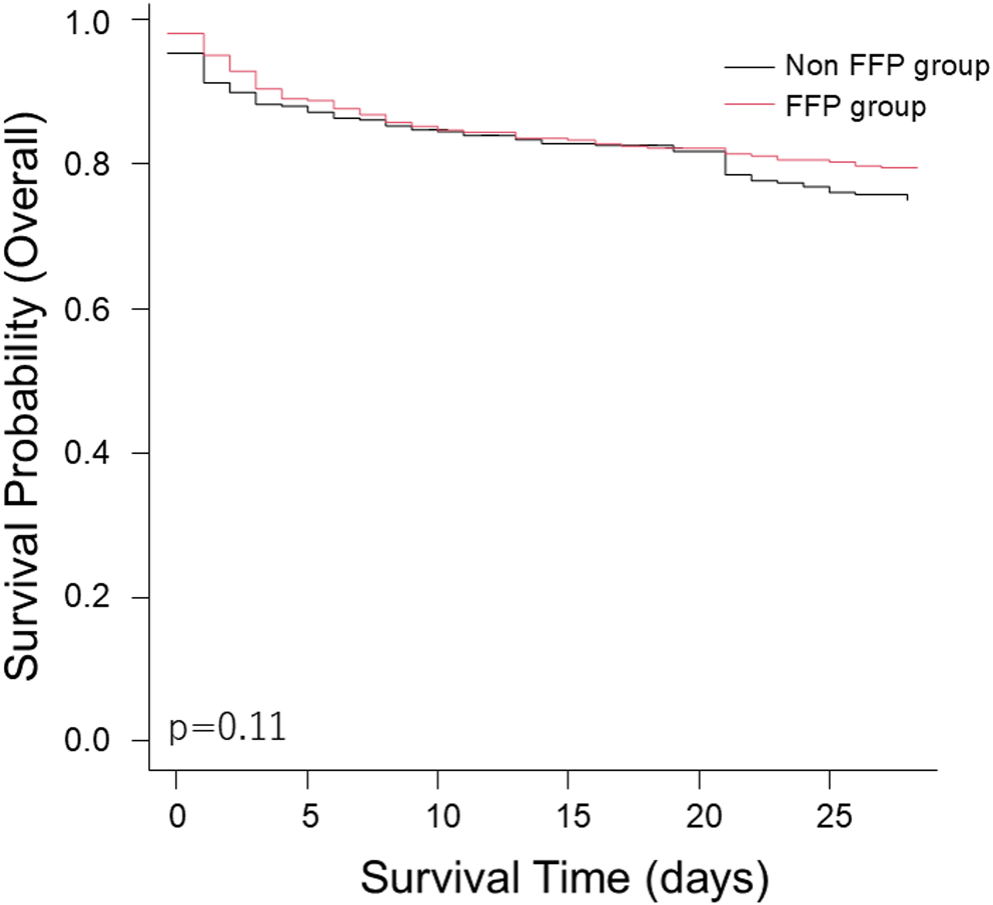
Kaplan–Meier survival curves for 28-day mortality in patients with ITBI, stratified by FFP administration. FFP, fresh frozen plasma; ITBI, isolated traumatic brain injury

**Fig. 4:**
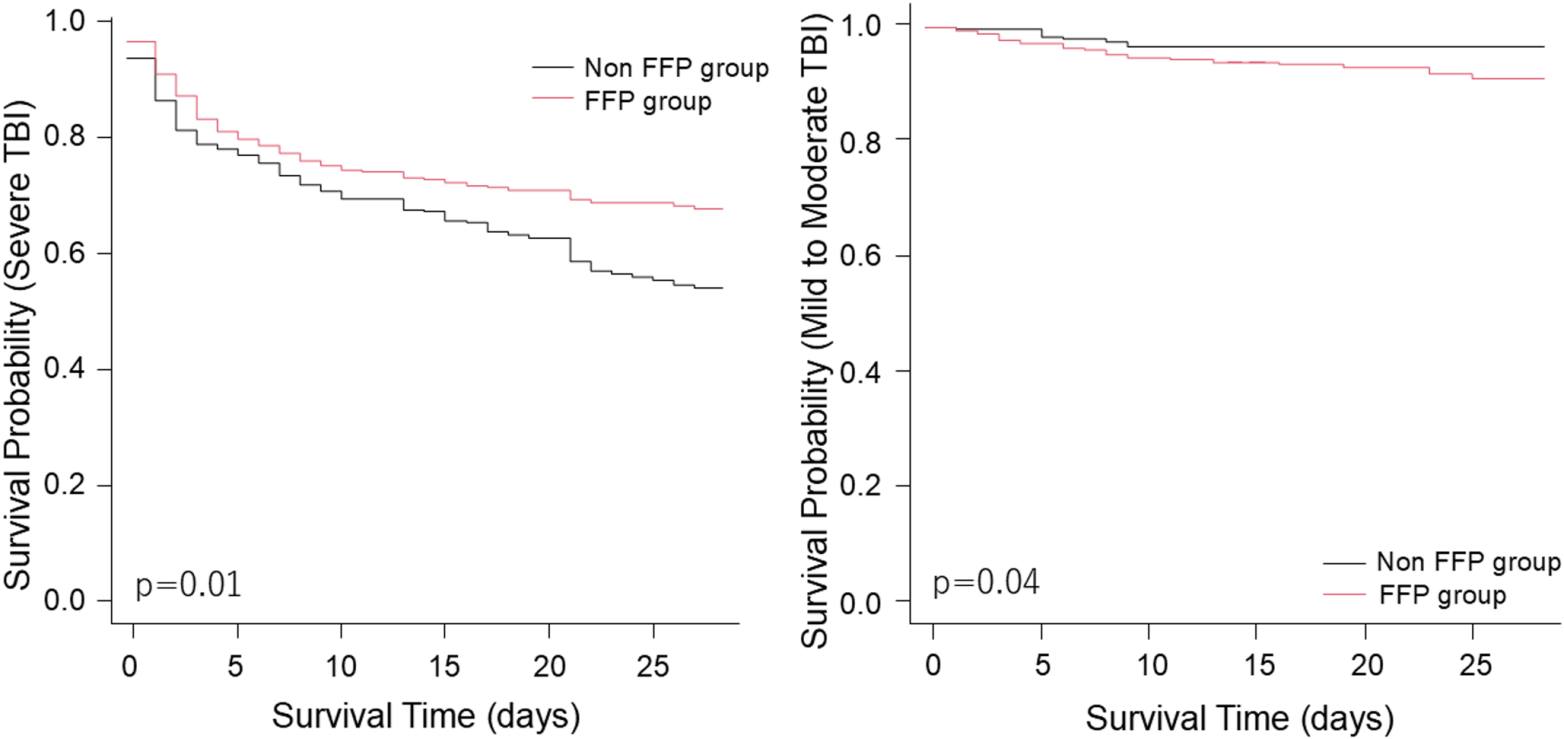
Kaplan–Meier survival curves for 28-day mortality stratified by FFP administration in patients with severe and mild-to-moderate ITBI. FFP, fresh frozen plasma; ITBI, isolated traumatic brain injury

Kaplan–Meier analysis showed no overall difference in 28-day survival between the groups in the matched cohort (Fig. 3). However, when stratified by TBI severity, survival curves diverged in the mild-to-moderate subgroup, with lower survival in patients receiving FFP. In contrast, in patients with severe TBI subgroup, the FFP group showed significantly higher survival (*p* = 0.01) (Fig. 4).

Sensitivity analyses yielded results consistent with the primary analysis. After excluding patients who died within 24 h, FFP was not associated with 28-day mortality in the overall cohort (OR 0.98, 95% CI 0.69–1.40, *p* = 0.93), whereas a significant increase in mortality was observed in the mild-to-moderate TBI subgroup (OR 3.50, 95% CI 1.28–9.57, *p* = 0.02) but not in the severe subgroup (OR 0.82, 95% CI 0.54–1.24, *p* = 0.35), with a significant interaction between subgroups (p for interaction < 0.01). In analyses including patients with adjustment for RBC transfusion, no significant association was observed in the overall cohort (OR 1.22, 95% CI 0.96–1.56, *p* = 0.10), while increased mortality persisted in the mild-to-moderate subgroup (OR 2.49, 95% CI 1.43–4.32, *p* < 0.01) but not in the severe subgroup (OR 1.07, 95% CI 0.80–1.43, *p* = 0.66), with a significant interaction (p for interaction < 0.01).

## Discussion

This study investigated the association between FFP administration and outcomes in patients with isolated TBI, focusing on severity according to GCS scores on arrival. We found that FFP administration was not associated with a reduction in the 28-day mortality rate in patients with isolated TBI. Notably, the subgroup analysis indicated that FFP administration was associated with higher 28-day mortality in patients with mild-to-moderate TBI and lower mortality in those with severe TBI. These findings suggest potential heterogeneity in the association between FFP administration and outcomes across GCS-defined TBI severity. However, given the absence of coagulation data and other key clinical variables in this dataset, this finding should be interpreted cautiously because these results may reflect FFP use as a marker of clinical deterioration rather than a direct causal relationship.

Both the timing of FFP administration and appropriate patient selection may be important factors when evaluating its impact on outcomes in TBI. To our knowledge, no studies have reported a clear benefit of FFP in isolated TBI with GCS ≤ 8. Secondary analyses of the PAMPer trial reported improved survival with prehospital plasma in patients with TBI, particularly those with GCS ≤ 8 [25]. In addition, a recent study in pediatric patients with severe TBI defined by AIS suggested that early plasma administration was associated with reduced 4-hour mortality, with patients receiving plasma presenting with markedly low GCS scores (median 3 [IQR 3–4]) [26]. Although these findings were derived from polytrauma populations with TBI, these reports suggest that potential benefits of FFP in TBI may be limited to specific subgroups. In our study, a potential benefit was observed in patients with isolated TBI and GCS ≤ 8, which may indicate a subgroup in whom FFP administration could be clinically relevant.

Our study also revealed that FFP administration was associated with improved 24-hour mortality. This may be explained by differences in the underlying pathophysiology across time phases after injury. In the early postinjury period, particularly within the first few hours after injury when fibrinogen levels are lowest, trauma-induced coagulopathy is most pronounced [3]. In this phase, FFP administration may contribute to hemostatic stabilization, potentially limiting hematoma expansion and early clinical deterioration, thereby reducing early mortality. However, later mortality in TBI is more influenced by secondary brain injury [27], extracranial complications [28], and multiple organ dysfunction [29], which may not be directly modified by early FFP administration. In fact, FFP administration in our study was associated with a higher rate of overall complications. Previous study including patients with both TBI and polytrauma have suggested that early plasma administration may be associated with improved short-term survival, particularly 4 h after injury [26], although consistent long-term benefit has not been demonstrated. Taken together, these findings suggest that the potential benefit of FFP may be limited to the early phase after injury and may not translate into improved long-term outcomes.

One of the strengths of our study is the careful selection of the study population. Our study specifically included patients with isolated TBI and excluded those who received RBC transfusions within 24 h, allowing us to assess the independent association of FFP administration. Another notable feature of our study was the inclusion of all TBI severity types, including mild-to-moderate cases. Given that FFP may be administered to this subgroup, evaluating its impact on non-severe TBI is clinically relevant. Therefore, our findings may offer insights into the potential benefits and risks of FFP administration across the full spectrum of TBI severity.

Future investigations should explore whether early intervention could improve clinical outcomes. Moreover, prospective multicenter studies and RCTs focusing on the timing of FFP administration and severity stratification based on GCS are warranted, especially in mild-to-moderate TBI. Ongoing RCTs, such as the FIT-BRAIN (FFP In Traumatic BRAin INjury) trial [30], which aims to evaluate the efficacy of FFP in patients with moderate-to-severe TBI, are expected to provide valuable evidence to inform future clinical practice.

This study has some limitations. First, its retrospective observational design may have introduced unmeasured confounding factors that could not be fully accounted for despite the use of PSM. In particular, residual confounding by indication may be present, as FFP may have been preferentially administered to patients who deteriorated after initial presentation rather than causing the observed associations. Second, coagulation variables at admission were not available in the JTDB, and specific indications for FFP administration were not recorded. Therefore, we could not determine whether FFP was appropriately administered. Third, the JTDB does not record the exact timing of FFP administration within the 24-hour window. Consequently, the exposure definition is unable to distinguish between early resuscitative use, pre-operative coagulopathy correction, and late rescue administration in response to clinical deterioration, each representing mechanistically distinct indications with potentially different effects on outcome. Moreover, data on hematoma burden, pupil reactivity, and the timing of intubation or sedation were not recorded in the registry, which may have influenced the assessment of TBI severity and the decision to administer FFP. Fourth, the registry did not specify the cause of death, limiting our ability to assess whether mortality was directly attributable to hemorrhage, brain injury, or other factors. Additionally, it was unclear whether the deaths were due to brain death or occurred after the decision to withhold or withdraw life-sustaining treatment. Furthermore, selection bias may have influenced the results, particularly in the severe TBI subgroup, as FFP may have been preferentially administered to patients perceived as having a realistic chance of recovery. This cannot be fully corrected by propensity score matching. Finally, data on neurological outcomes at 6 months after trauma were not available, precluding assessment of long-term functional prognosis.

## Conclusions

In this nationwide cohort study on patients with isolated TBI, FFP administration was not associated with a reduction in 28-day mortality. Subgroup analyses suggested that the association between FFP administration and outcomes may vary according to GCS-defined TBI severity, although these findings should be interpreted with caution due to potential residual confounding. Further prospective studies are needed to clarify the optimal indications for FFP administration in TBI.

## Supplementary Information

Below is the link to the electronic supplementary material.

Supplementary Material 1

Supplementary Material 2

Supplementary Material 3

## Data Availability

The datasets used and/or analyzed in the current study are available from the corresponding author upon reasonable request.

## Abbreviations

AIS: Abbreviated Injury Scale
ARDS: Acute respiratory distress syndrome
CCI: Charlson Comorbidity Index
CI: Confidence interval
FFP: Fresh frozen plasma
GCS: Glasgow Coma Scale
HR: Heart rate
IQR: Interquartile range
ISS: Injury Severity Score
JTDB: Japan Trauma Data Bank
OR: Odds ratio
RBC: Red blood cell
RCT: Randomized controlled trial
SBP: Systolic blood pressure
SMD: Standardized mean difference
STROBE: Strengthening the Reporting of Observational Studies in Epidemiology
TBI: Traumatic brain injury
TXA: Tranexamic acid
