# Supplementary figures and images for: Association between fresh frozen plasma transfusion and mortality stratified by Glasgow Coma Scale scores in isolated traumatic brain injury: a nationwide cohort study in Japan
Source: Eur J Trauma Emerg Surg. 2026 Jul 1;52(1):211. doi: 10.1007/s00068-026-03249-7 (PMC13323265; doi:10.1007/s00068-026-03249-7)

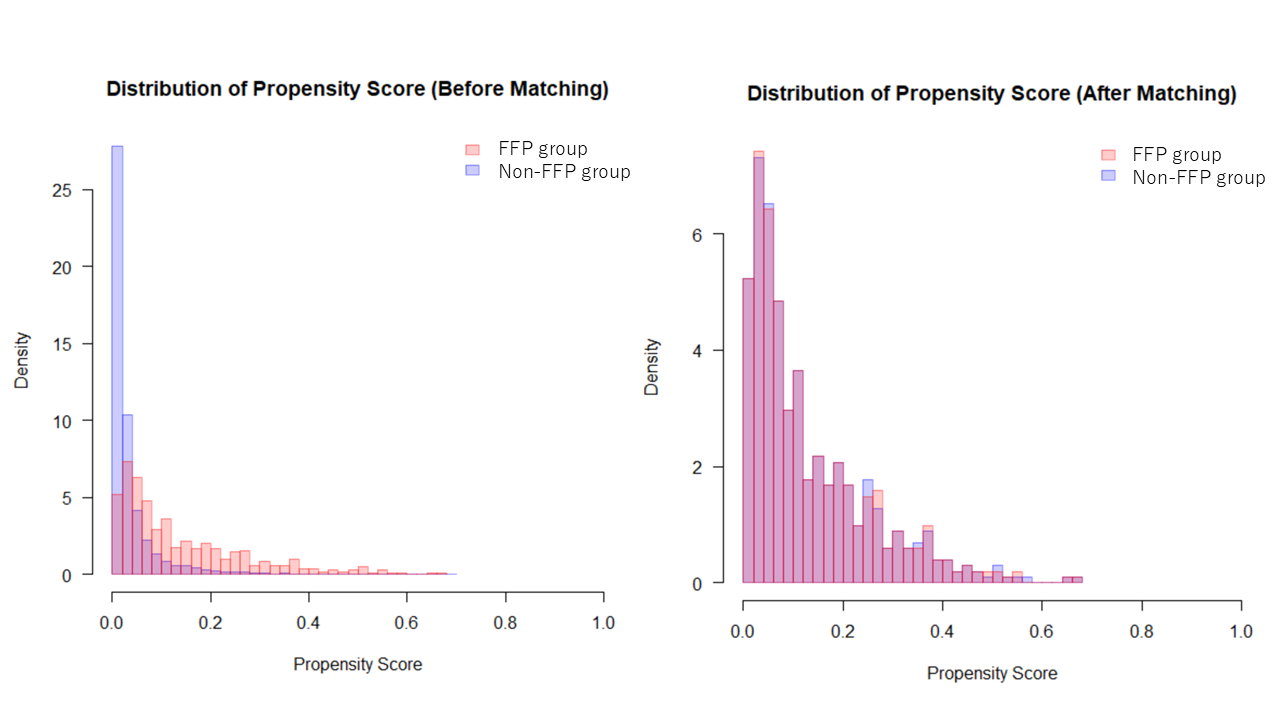

Supplement: Supplementary file 1 — Supplementary Material 1 [file 68_2026_3249_MOESM1_ESM.tif]
