# Supplementary material for: Association between fresh frozen plasma transfusion and mortality stratified by Glasgow Coma Scale scores in isolated traumatic brain injury: a nationwide cohort study in Japan
Source: Eur J Trauma Emerg Surg. 2026 Jul 1;52(1):211. doi: 10.1007/s00068-026-03249-7 (PMC13323265; doi:10.1007/s00068-026-03249-7)
Supplement: Supplementary file 2 — Supplementary Material 2 [file 68_2026_3249_MOESM2_ESM.docx]

**Supplementary Figure Legends**

**Supplementary Fig. 1 Propensity score distributions before and after matching**

FFP, fresh frozen plasma
