# Supplementary material for: Association between fresh frozen plasma transfusion and mortality stratified by Glasgow Coma Scale scores in isolated traumatic brain injury: a nationwide cohort study in Japan
Source: Eur J Trauma Emerg Surg. 2026 Jul 1;52(1):211. doi: 10.1007/s00068-026-03249-7 (PMC13323265; doi:10.1007/s00068-026-03249-7)
Supplement: Supplementary file 3 — Supplementary Material 3 [file 68_2026_3249_MOESM3_ESM.docx]

**Table S1. Lists of hemorrhagic traumatic brain injury code of abbreviated injury scale 2005**

| Epidural hematoma | Subdural hematoma | Subarachnoid hemorrhage | Intraparenchymal hemorrhage |
| --- | --- | --- | --- |
| 140414.3 | 140438.3 | 140695.3 | 140426.3 |
| 140418.4 | 140442.4 |  | 140430.4 |
| 140422.5 | 140446.5 |  | 140434.5 |
| 140630.3 | 140650.3 |  | 140638.3 |
| 140632.4 | 140651.3 |  | 140645.4 |
| 140634.5 | 140652.4 |  | 140640.4 |
| 140636.5 | 140654.4 |  | 140647.3 |
|  | 140656.5 |  | 140649.4 |
|  | 140655.5 |  | 140646.5 |
|  |  |  | 140648.5 |
|  |  |  | 140641.5 |

**Table S2. Characteristics of overall patients.**

| Variables | Overall (n=12,480) |
| --- | --- |
| Age, y, median (IQR) | 72 [55, 82] |
| Sex, male, n (%) | 8,309 (66.6) |
| Charlson score, median (IQR) | 0 [0, 1] |
| Alcohol use, n (%) | 3,608 (28.9) |
| Blunt injury, n (%) | 12,369 (99.1) |
| Injury type, n (%) |  |
| Motor vehicle crash | 468 (3.8) |
| Motorcycle | 768 (6.2) |
| Pedestrian | 962 (7.7) |
| Bicycle | 1,269 (10.2) |
| Falling from height | 647 (5.2) |
| Falling at grounding level | 4,842 (38.8) |
| Head Injury Type |  |
| Hemorrhagic TBI |  |
| Epidural hematoma (EDH) | 1,331 (10.7) |
| Subdural hematoma (SDH) | 8,179 (65.5) |
| Subarachnoid hemorrhage (SAH) | 790 (6.3) |
| Intraparenchymal hematoma (IPH) | 355 (2.8) |
| Non-hemorrhagic TBI | 2,846 (22.8) |
| TBI severity |  |
| Severe | 2,752 (22.1) |
| Mild to moderate | 9,728 (77.9) |
| Vital signs |  |
| SBP, mmHg, median (IQR) | 150 [131, 173] |
| HR, bpm, median (IQR) | 84 [73, 96] |
| GCS, median (IQR) | 14 [10, 15] |
| Head AIS, median (IQR) | 3 [3, 4] |
| ISS, median (IQR) | 14 [9, 20] |
| FFP transfusion, n (%) | 513 (4.1%) |
| FFP transfusion volume in FFP group, units, median [IQR] | 4 [4, 8] |
| TXA, n (%) | 3,185 (25.5) |
| Craniotomy, n (%) | 1,109 (8.9) |
| Mortality |  |
| 24 hours, n (%) | 526 (4.2) |
| 28 days, n (%) | 1,423 (11.4) |
| Length of stay in hospital, median (IQR) | 14 [5, 28] |
| Overall complication, n (%) | 3,710 (29.7) |

FFP, fresh frozen plasma; ASMD, absolute standardized mean difference; SBP, systolic blood pressure; HR, heart rate; GCS, Glasgow coma scale; AIS, abbreviated injury scale; ISS, injury severity scale, TXA, tranexamic acid

Hemorrhagic TBI categories (EDH, SDH, SAH, and IPH) were not mutually exclusive; in patients with multiple types of intracranial hemorrhage, each hemorrhage type was counted separately.

Non-hemorrhagic TBI includes diffuse axonal injury, diffuse brain swelling, and selected cases of pneumocephalus meeting AIS ≥3 criteria. Simple concussion without intracranial injury was not included.
